# Risk factors for endometrial cancer incidence and regional and ethnic disparities in the USA

**DOI:** 10.7189/jogh.16.04368

**Published:** 2026-10-23

**Authors:** Victor Adekanmbi, Fangjian Guo, Jiefei Wang, Angela Zhang, Christine H Wang, Thao N. Hoang, Itunu Sokale, Olalekan Uthman, Abbey B. Berenson

**Affiliations:** 1 Center for Interdisciplinary Research in Women's Health, School of Medicine, The University of Texas Medical Branch, Galveston, Texas, USA; 2 Department of Obstetrics and Gynecology, The University of Texas Medical Branch, Galveston, Texas, USA; 3 Department of Biostatistics and Data Science, The University of Texas Medical Branch, Galveston, Texas, USA; 4 School of Medicine, The University of Texas Medical Branch, Galveston, Texas, USA; 5 Department of Medicine, Section of Epidemiology and Population Sciences, Baylor College of Medicine, Houston, Texas, USA; 6 Dan L. Duncan Comprehensive Cancer Center, Baylor College of Medicine, Houston, Texas, USA; 7 Division of Health Sciences, Warwick-Centre for Applied Health Research and Delivery, The University of Warwick, Coventry, UK

**Keywords:** endometrial cancer, mixed effect analysis, individual factors, contextual factors, racial and ethnic disparities, USA

## Abstract

**Background:**

Endometrial cancer (EC) is the most common gynaecologic cancer in the USA and a major cause of cancer-associated mortality among women. We aimed to identify the independent contributions of individual- and contextual-level factors to EC incidence.

**Methods:**

We conducted a longitudinal retrospective cohort study using anonymised data from women aged 18 years and above without EC included in the All of Us Research database before 1 January 2017. We followed participants from study entry until October 2023 (median follow-up = 6.6 years). We used mixed-effects Cox regression models to examine data from individuals nested within ZIP-code areas and states, analysing individual-level risk factors and neighbourhood-level socioeconomic predictors in addition to adjusting for area-level clustering.

**Results:**

Of 81,171 eligible women followed for 496,709 person-years, 778 (0.96%) developed EC. The risk of EC risk approximately thrice as high among women aged 50–59 years (adjusted hazard ratio (aHR) = 2.92; 95% confidence interval (CI) = 2.20–3.88), 60–69 years (aHR = 2.97; 95% CI = 2.21–4.00), and ≥70 years (aHR = 2.86; 95% CI = 2.02–4.06) than among women under 40 years of age. Non-Hispanic White women had a 44% higher risk than non-Hispanic Black women (aHR = 1.44; 95% CI = 1.15–1.80). Obesity was associated with more than twice the risk of EC (aHR = 2.16; 95% CI = 1.85–2.52), while diabetes was associated with a 27% higher risk (aHR = 1.27; 95% CI = 1.06–1.51).

**Conclusion:**

Individual-level factors, particularly age and obesity, were the main predictors of EC incidence. Prevention efforts should prioritise obesity reduction, age-specific screening awareness, and lifestyle modifications.

Endometrial cancer (EC) is the most common gynaecologic malignancy in the USA and other high-income countries, and is an increasingly important contributor to cancer-related morbidity and mortality among women [1–3]. It accounts for approximately 40% of all gynaecologic cancers diagnosed annually in the USA, with more than 66,000 new cases and over 13,000 deaths expected each year [3]. Although EC historically has been associated with more favourable outcomes than other gynaecologic malignancies, the related mortality rates have increased over the past two decades, contrasting trends observed in many other cancers [1,2].

In contrast to ovarian cancer, EC often presents with early clinical symptoms, most commonly abnormal uterine bleeding, facilitating diagnosis at an early and localised stage. Consequently, five-year survival for early-stage disease exceeds 80% [4]. However, this survival advantage is not uniform across patient groups. Women diagnosed with advanced-stage disease or high-risk histologic subtypes, including serous and clear cell carcinomas, have substantially poorer outcomes despite advances in surgical management and adjuvant therapies [4–6]. Moreover, improvements in early detection have not translated into proportional reductions in population-level mortality, suggesting that additional biological and contextual factors influence disease outcomes [2,5].

The aetiology of EC is multifactorial and strongly linked to hormonal and metabolic dysregulation. Prolonged exposure to oestrogen unopposed by progesterone is a central pathogenic mechanism, underlying several risk factors, including obesity, diabetes mellitus, polycystic ovarian syndrome, early menarche, late menopause, and nulliparity, which increase disease risk [7–9]. Obesity is consistently identified as the most influential modifiable risk factor, conferring two to five times the risk through mechanisms involving peripheral oestrogen production, insulin resistance, and chronic inflammation [7,9]. Inherited cancer predisposition syndromes, particularly Lynch syndrome, also contribute to EC risk, especially among younger women [10]. Despite these established associations, the natural history of EC and the interactions between biological, behavioural, and environmental determinants remain incompletely characterised [8,11].

Most epidemiologic studies evaluating predictors of EC incidence in the USA have focused primarily on individual-level compositional factors such as age, body mass index (BMI), reproductive history, and genetic susceptibility. Although informative, this approach often overlooks the contribution of contextual and area-level factors, including neighbourhood socioeconomic conditions, access to healthcare, and urban-rural residential patterns. Socioecological determinants of health have, therefore, received increasing attention because they may directly and indirectly influence cancer risk, detection, and outcomes, particularly for malignancies closely linked to metabolic health [12–14].

Few population-based studies have examined the independent effects of individual-, neighbourhood- and state-level contextual factors on EC incidence in the USA within a single analytical framework. Analyses limited to individual-level data may be susceptible to the atomistic fallacy because they do not account for shared environmental and structural exposures. Conversely, studies relying solely on aggregate contextual measures may be subject to the ecological fallacy because they obscure individual-level heterogeneity. Evaluating individual and contextual determinants simultaneously may provide a more comprehensive understanding of EC risk, while addressing key methodological limitations in existing literature.

We aimed to assess the independent effects of individual-, neighbourhood-, and state-level contextual factors on predicting EC incidence using large-scale, population-based longitudinal data from the USA.

## METHODS

### Data source

We used data from All of Us (AoU), a nationwide research initiative that provides access to de-identified electronic health records (EHRs), survey responses, physical measurements, and genomic data from more than 633,000 participants across the USA. AoU releases a curated data repository on an annual basis. For this study, we used Version 8, the most recent release available at the time of the analysis, which contains data collected up to 1 October 2023 [15]. The AoU defines two tiers of data access: the Registered Tier, which contains EHR data and survey data, and the Controlled Tier, which additionally includes genomic data and geographic information. We used data from the Controlled Tier for this study.

We report our study per the STROBE guidelines [16] and confirm it aligns with the GRABDROP guidelines for secondary data analyses [17] (Tables S1 and S2 in the **Online Supplementary Document**).

### Study population

We defined the study period as 1 January 2017 to 31 October 2023 and used the start date as the index date for the cohort. Eligible participants were aged 18 years or older at cohort entry, self-identified as female, and had complete information on age, race, and ethnicity. Participants were also required to have at least one recorded healthcare visit during the year before cohort entry and at least one visit after the cohort entry to ensure sufficient longitudinal data.

We identified EC using Observational Medical Outcomes Partnership standard condition concepts for malignant neoplasms of the endometrium, including concept identifier 4048225. We excluded participants with any history of EC prior to the index date or a history of hysterectomy to restrict the cohort to those at risk of incident EC.

### Variables and measures

The primary outcome was incident EC, defined as the first recorded EC diagnosis after cohort entry. We calculated time to event from the index date to the first recorded EC diagnosis, the participant’s last recorded clinical visit, or the study end date of 31 October 2023, whichever occurred first. Participants without recorded EC diagnosis during follow-up were considered cancer-free and were censored at the end of the study period (31 October 2023).

### Individual-level variables

We retrieved several individual-level variables from the AoU data set, including demographic characteristics, socioeconomic factors, and medical history. Demographic variables included age, race, and ethnicity, with age calculated at the index date based on each participant's date of birth. Socioeconomic factors were sourced from the survey data and included nativity, employment status, smoking status, annual income, and marital status. We identified medical history, including diabetes and obesity, prior to the index date from EHR data. We derived anthropometric measures from clinical measurements (height, weight, and standardised BMI fields). We defined obesity as BMI ≥ 30 kg/m^2^ and analysed it as a binary variable. As AoU does not capture combined oral contraceptive use, we initially considered a history of progesterone-only prescriptions as a proxy for hormonal exposure. However, the prescription data did not distinguish contraceptive from therapeutic use. Moreover, progestins are commonly prescribed for abnormal uterine bleeding, a frequent symptom of EC, potentially conflating a protective exposure with a marker of the underlying disease. We therefore excluded it from the primary models and reported it only in a sensitivity analysis.

### Neighbourhood-level variables

We obtained neighbourhood-level variables at cohort entry from the AoU Socioeconomic Status table, which was derived from the 2017 American Community Survey one-year estimates. We linked these data to participants at the ZIP code level. We categorised the percentage of high school graduates and the percentage of households receiving assisted income into quintiles (1–5) based on their distributions within the AoU cohort. We classified geographic region of residence as Northeast, Midwest, West, or South according to each participant’s ZIP code.

### Statistical analysis

We summarised categorical variables as frequencies and percentages, and continuous variables as medians with interquartile ranges. We used Kaplan-Meier curves to visualise cumulative EC incidence during the follow-up period. We applied mixed-effects Cox proportional hazards regression models to examine individual- and contextual-level predictors of EC incidence simultaneously and account for the hierarchical structure of individuals nested within ZIP code areas and states. This approach addresses the methodological limitations of standard Cox regression, which assumes independent observations and may produce biased estimates when applied to clustered data.

We fitted four progressive models. Model 1, the null model, contained no fixed-effect predictors and estimated baseline geographic clustering. Model 2 added individual-level covariates, while model 3 added neighbourhood-level variables only. Model 4, the full model, included all individual- and neighbourhood-level covariates simultaneously. We specified random effects at two levels: ZIP code area and state.

We summarised variance components using the variance partition coefficient (VPC) and the median hazard ratio (MHR). We calculated the VPC on the Gumbel latent scale appropriate for a proportional hazards model (residual variance = π^2^/6), with both random effects in the denominator. We calculated the MHR as MHR = exp(√2 σ Φ^−1^(0.75)) [18]. Because the null value of a variance component lies on the boundary of the parameter space, we referred likelihood ratio tests for random effects to a 50:50 mixture of χ^2^ 0 and χ^2^ 1 [19]. We assessed the proportional hazards assumptions using Schoenfeld residuals and compared model fit using the Akaike information criterion (AIC), with lower values indicating better fit.

We presented results as adjusted hazard ratios (aHR) with 95% confidence intervals (CI) and defined statistical significance as two-sided *P* < 0.05. Because the proportional hazards assumption did not hold for obesity, we characterised the time dependence of this association by splitting follow-up at three years and estimating period-specific hazard ratios, with the baseline hazard stratified by period. To assess potential outcome misclassification arising from EHR-derived diagnostic coding, we repeated the full model using progressively stricter case definitions that required at least two, followed by at least three, recorded EC condition concepts. To quantify the potential influence of unmeasured confounding arising from established EC risk factors not captured in AoU, we computed E-values for each principal estimate [20]. The E-value represents the minimum strength of association, on the risk-ratio scale, that an unmeasured confounder would need to have with both the exposure and the outcome, conditional on the measured covariates, to fully explain an observed association.

We conducted all analyses using *R*, version 4.6.1 (R Core Team, Vienna, Austria), with the ‘coxme’ package.

## RESULTS

### Descriptive statistics

Of 294,244 female participants who were ≥18 years of age at enrolment in the AoU, we included 81,171 (28.8%) who met all the eligibility criteria (**Figure 1**). We excluded 207,302 participants (70.5%) who did not have at least one clinical visit during the year preceding the index date and at least one visit during or after the study period; 1,228 (0.4%) with a documented diagnosis of EC before the index date; 827 (0.3%) who were missing geographic variables required for the mixed-effects modelling. Among otherwise eligible women, 3,744 were excluded because of a prior hysterectomy. Exclusion differed by race and ethnicity: 2,662 of 61,252 non-Hispanic White women (4.4%) and 925 of 19,097 non-Hispanic Black women (4.8%) were excluded. Of the included 81,171 women who were followed up from 1 January 2017 or the day they entered the study to October 2023, 778 (0.96%) had incident EC.

**Figure 1:**
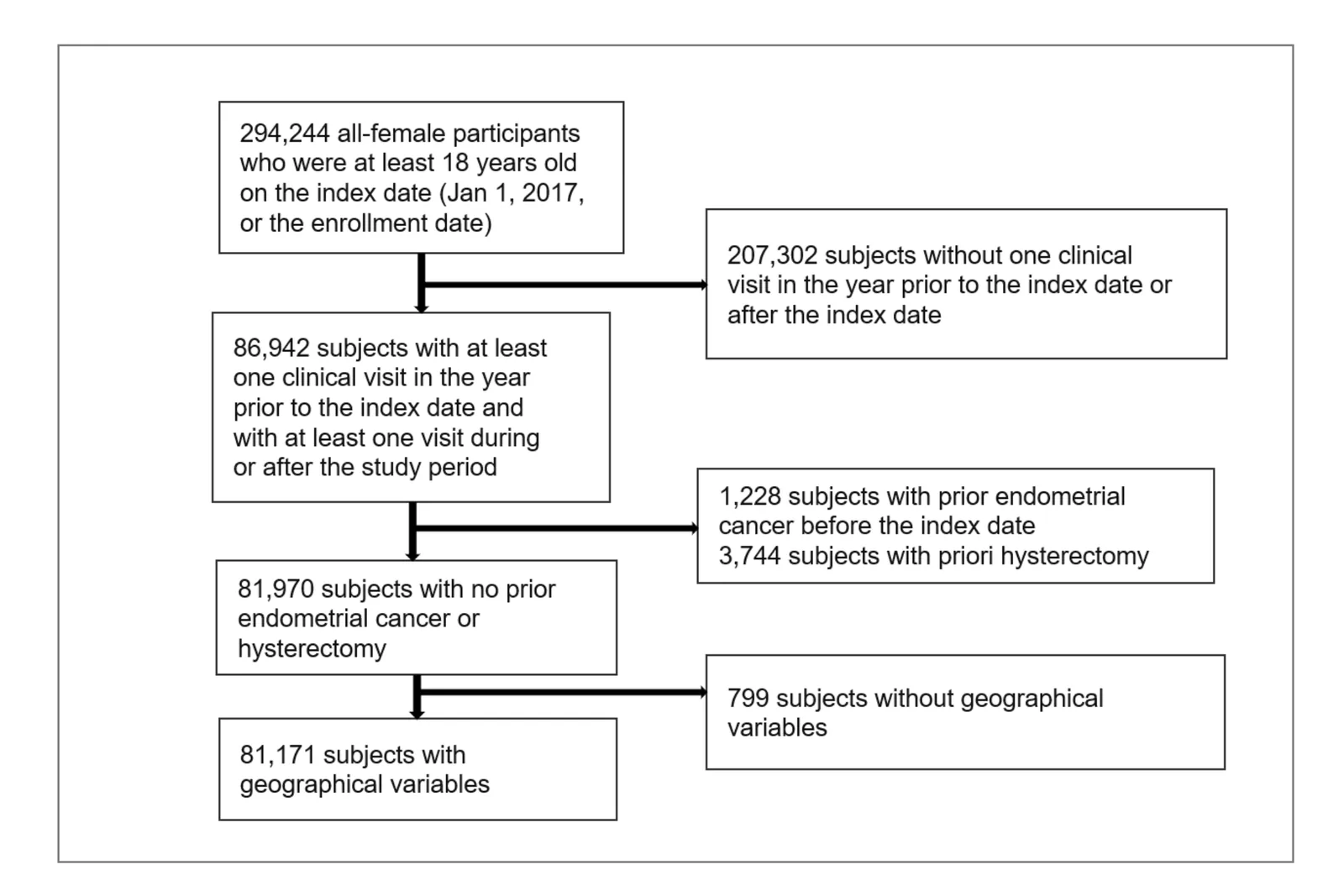
Flow diagram of the participant inclusion and exclusion procedure.

The estimated survival probability dropped from 1.0 at baseline to approximately 0.995 at 1,095 days of follow-up, as indicated by the Kaplan-Meier survival curve and its 95% CI. The number of participants at risk decreased from 81,171 at baseline to 80,883 at 1 year, 79,579 at 2 years, 71,168 at 5 years, and down to 64,959 at the end of follow-up (approximately 7 years). The median follow-up time for the entire cohort was 2,419 days (**Figure 2**).

**Figure 2:**
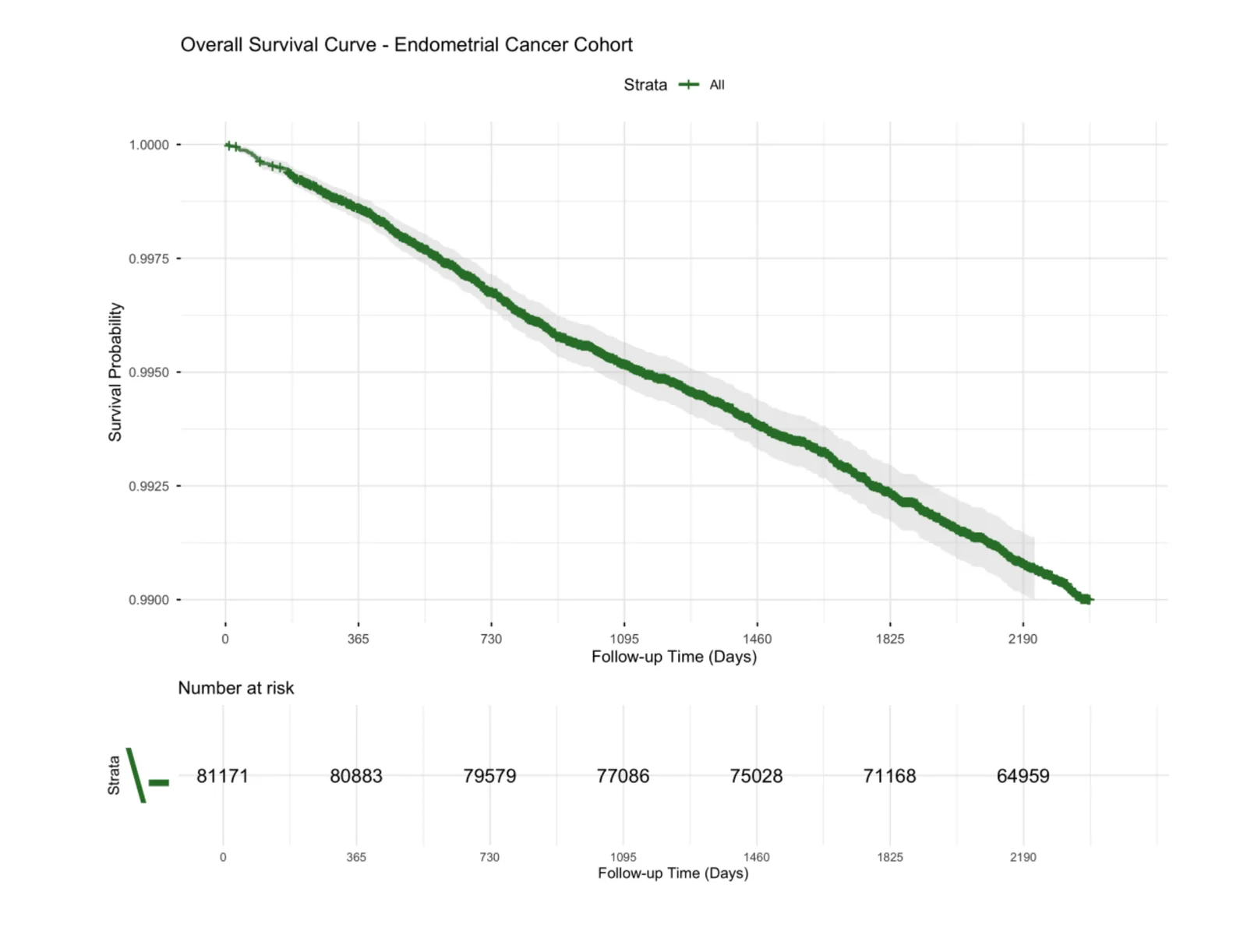
Kaplan-Meier survival plot with 95% confidence interval.

Approximately 35% of individuals who developed EC were aged 60–69 years, whereas approximately 9% were aged 40 years or below. The incidence of EC was significantly higher among participants who were obese, employed, and had diabetes. It was also highest among participants who were non-Hispanic White, those earning USD 50,000 or less per year, and those residing in neighbourhoods in the Northeast region (**Table 1**).

**Table 1:** Characteristics of the study population*

	Overall	No event	Event	*P-*value
**Follow-up time in days, Mdn (IQR)**	2,419 (2,280, 2,452)	2,420 (2,290, 2,452)	1,107 (568, 1,769)	<0.001
**Age group in years**				<0.001
<40	19,072 (23.5)	19,004 (23.6)	68 (8.7)	
40–49	12,409 (15.3)	12,289 (15.3)	120 (15.4)	
50–59	16,598 (20.4)	16,407 (20.4)	191 (24.6)	
60–69	22,099 (27.2)	21,826 (27.1)	273 (35.1)	
≥70	10,993 (13.5)	10,867 (13.5)	126 (16.2)	
**Country of birth**				>0.900
Foreign-born	5,654 (7.0)	5,600 (7.0)	54 (6.9)	
USA-born	75,517 (93.0)	74,793 (93.0)	724 (93.1)	
**Employment status**				<0.001
Employed	39,739 (49.0)	39,416 (49.0)	323 (41.5)	
Retired	23,587 (29.1)	23,295 (29.0)	292 (37.5)	
Unemployed	17,845 (22.0)	17,682 (22.0)	163 (21.0)	
**Race/ethnicity**				0.011
Black	18,172 (22.4)	18,030 (22.4)	142 (18.3)	
Hispanic	1,541 (1.9)	1,532 (1.9)	9 (1.2)	
Other/unknown	2,868 (3.5)	2,842 (3.5)	26 (3.3)	
White	58,590 (72.2)	57,989 (72.1)	601 (77.2)	
**Marital status**				0.600
Married	44,341 (54.6)	43,908 (54.6)	433 (55.7)	
Unmarried	36,830 (45.4)	36,485 (45.4)	345 (44.3)	
**Obesity**				<0.001
Non-obese	46,872 (57.7)	46,556 (57.9)	316 (40.6)	
Obese	34,299 (42.3)	33,837 (42.1)	462 (59.4)	
**Smoking status**				0.800
Ex-smoker/current smoker	30,864 (38.0)	30,572 (38.0)	292 (37.5)	
Non-smoker	30,864 (38.0)	30,572 (38.0)	292 (37.5)	
**Diabetes**	12,424 (15.3)	12,247 (15.2)	177 (22.8)	<0.001
**Pelvic inflammatory disease**	15,454 (19.0)	15,300 (19.0)	154 (19.8)	0.600
**Endometriosis**	1,869 (2.3)	1,845 (2.3)	24 (3.1)	0.200
**Hormone replacement therapy**	438 (0.5)	434 (0.5)	4 (0.5)	>0.900
**Peutz-Jeghers syndrome**	152 (0.2)	149 (0.2)	3 (0.4)	0.400
**Annual income in USD**				0.700
≤50,000	36,397 (44.8)	36,056 (44.8)	341 (43.8)	
50,000–75,000	10,215 (12.6)	10,124 (12.6)	91 (11.7)	
75,000–100,000	8,286 (10.2)	8,201 (10.2)	85 (10.9)	
≥100,000	26,273 (32.4)	26,012 (32.4)	261 (33.5)	
**ZIP: high school education**				0.001
Quintile 1	16,370 (20.2)	16,205 (20.2)	165 (21.2)	
Quintile 2	16,241 (20.0)	16,115 (20.0)	126 (16.2)	
Quintile 3	16,260 (20.0)	16,126 (20.1)	134 (17.2)	
Quintile 4	16,145 (19.9)	15,954 (19.8)	191 (24.6)	
Quintile 5	16,155 (19.9)	15,993 (19.9)	162 (20.8)	
**ZIP: assisted income**				0.044
Quintile 1	17,546 (21.6)	17,377 (21.6)	169 (21.7)	
Quintile 2	14,965 (18.4)	14,799 (18.4)	166 (21.3)	
Quintile 3	16,597 (20.4)	16,448 (20.5)	149 (19.2)	
Quintile 4	15,864 (19.5)	15,738 (19.6)	126 (16.2)	
Quintile 5	16,199 (20.0)	16,031 (19.9)	168 (21.6)	
**ZIP: uninsured**				<0.001
Quintile 1	17,498 (21.6)	17,304 (21.5)	194 (24.9)	
Quintile 2	15,047 (18.5)	14,913 (18.6)	134 (17.2)	
Quintile 3	17,166 (21.1)	16,976 (21.1)	190 (24.4)	
Quintile 4	16,144 (19.9)	16,031 (19.9)	113 (14.5)	
Quintile 5	15,316 (18.9)	15,169 (18.9)	147 (18.9)	
**ZIP: vacant housing**				<0.001
Quintile 1	16,389 (20.2)	16,223 (20.2)	166 (21.3)	
Quintile 2	16,113 (19.9)	15,970 (19.9)	143 (18.4)	
Quintile 3	16,820 (20.7)	16,660 (20.7)	160 (20.6)	
Quintile 4	16,177 (19.9)	15,979 (19.9)	198 (25.4)	
Quintile 5	15,672 (19.3)	15,561 (19.4)	111 (14.3)	
**Geographic region**				<0.001
Midwest	26,467 (32.6)	26,187 (32.6)	280 (36.0)	
Northeast	29,498 (36.3)	29,185 (36.3)	313 (40.2)	
South	15,680 (19.3)	15,573 (19.4)	107 (13.8)	
West	9,526 (11.7)	9,448 (11.8)	78 (10.0)	

*Presented as n (%) unless specified otherwise.

### Mixed effects analysis of the factors associated with risk of endometrial cancer

The full model (model 4; AIC = 17,222.4) showed meaningful improvement in fit over the null model (model 1; AIC = 17,411.9), indicating that combining individual- and neighbourhood-level factors improved the prediction of EC incidence. In contrast, the model containing only neighbourhood factors (model 3; AIC = 17,419.8) did not improve upon the null model, suggesting that contextual variables contributed to model fit only when combined with individual-level characteristics, rather than as independent predictors (**Table 3**).

**Table 3:** Measures of variation for random effects model and fit statistics*

	Model 1	Model 2	Model 3	Model 4
**State level**				
Variance	0.05	0.06	0.00	0.00
VPC (%)	1.4	1.7	0.0	0.0
MHR	1.23	1.25	1.00	1.00
Explained variance (%)†	ref	−16.7	100.0	100.0
**ZIP code level**				
Variance	0.04	0.04	0.00	0.00
VPC (%)	2.7	2.8	0.0	0.0
MHR	1.22	1.21	1.02	1.01
Explained variance (%)	ref	10.4	99.1	99.9
**Model fit statistics**				
AIC	17,411.9	17,212.0	17,419.8	17,222.4
df	0	21	19	40

aHR – adjusted hazard ratio, AIC – Akaike information criterion, CI – confidence interval, df – degrees of freedom, MHR – median hazard ratio, ref – reference, VPC – variance partition coefficient

*Model 1 was the null model; model 2 included individual-level factors; model 3 included neighbourhood-level factors; model 4 included individual- and neighbourhood-level factors.

†We computed VPC on the Gumbel latent scale (residual variance = π^2^/6) and MHR as exp(√2 × σ × Φ^−1^(0.75)). Explained variance represents the percentage change in the variance component relative to the null model; negative values indicate a reduction.

Participants’ age was the strongest predictor of incidence of EC after adjusting for all other factors. Women in all older age groups had significantly higher hazard of EC than women younger than 40 in the final model (model 4). Specifically, women aged 40–49 years had 2-fold increased risk of EC (aHR = 2.40; 95% CI = 1.78–3.24), while those aged 50–59 years (aHR = 2.92; 95% CI = 2.20–3.88), aged 60–69 years (aHR = 2.97; 95% CI = 2.21–4.00), and aged 70 years or older (aHR = 2.86; 95% CI = 2.02–4.06) all had a 3-fold increased risk (**Table 2**). Race and ethnicity showed significant associations, with non-Hispanic White women having a 44% higher risk of EC compared to non-Hispanic Black women (aHR = 1.44; 95% CI = 1.15–1.80). Similarly, women from other or unknown racial and ethnic groups had a 66% higher risk of EC than non-Hispanic Black women (aHR = 1.66; 95% CI = 1.02–2.70). Women with a history of diabetes had increased risk of EC (aHR = 1.27; 95% CI = 1.0–1.51), while women with obesity (**Table 2**) had a 2.16-fold increased risk (aHR = 2.11; 95% CI = 1.85-2.52).

**Table 2:** Multilevel Cox regression fixed-effects models for rates of endometrial cancer in the USA, aHR (95% CI)*

	Model 1	Model 2	Model 3	Model 4
**Individual-level factors**				
Age group in years				
*<40*	NA	1.00 (ref)	NA	1.00 (ref)
*40–49*	NA	2.39 (1.77–3.22)	NA	2.40 (1.78–3.24)
*50–59*	NA	2.89 (2.18–3.84)	NA	2.92 (2.20–3.88)
*60–69*	NA	2.95 (2.19–3.97)	NA	2.97 (2.21–4.00)
*≥70*	NA	2.85 (2.01–4.04)	NA	2.86 (2.02–4.06)
Nativity				
*USA-born*	NA	1.00 (ref)	NA	1.00 (ref)
*Foreign-born*	NA	0.98 (0.71–1.35)	NA	0.95 (0.69–1.31)
Employment status				
*Employed*	NA	1.00 (ref)	NA	1.00 (ref)
*Unemployed*	NA	1.02 (0.83–1.26)	NA	1.03 (0.84–1.27)
*Retired*	NA	1.10 (0.89–1.36)	NA	1.11 (0.89–1.37)
Race/ethnicity				
*Non-Hispanic Black*	NA	1 (ref)	NA	1 (ref)
*Non-Hispanic White*	NA	1.52 (1.22–1.89)	NA	1.44 (1.15–1.80)
*Other/unknown*	NA	1.72 (1.06–2.78)	NA	1.66 (1.02–2.70)
*Hispanic*	NA	1.04 (0.52–2.05)	NA	1.00 (0.50–1.98)
Marital status				
*Married*	NA	1.00 (ref)	NA	1.00 (ref)
*Unmarried*	NA	0.97 (0.83–1.15)	NA	0.99 (0.84–1.17)
Obesity				
*Non-obese*	NA	1.00 (ref)	NA	1.00 (ref)
*Obese*	NA	2.17 (1.86–2.53)	NA	2.16 (1.85–2.52)
Smoking status				
*Non-smoker*	NA	1.00 (ref)	NA	1.00 (ref)
*Ex-smoker/current smoker*	NA	1.07 (0.88–1.31)	NA	1.07 (0.87–1.30)
Diabetes (yes *vs.* no)	NA	1.27 (1.06–1.52)	NA	1.27 (1.06–1.51)
Pelvic inflammatory disease	NA	1.02 (0.85–1.23)	NA	1.00 (0.83–1.20)
Hormone replacement therapy	NA	0.73 (0.27–2.02)	NA	0.75 (0.27–2.06)
Endometriosis	NA	1.24 (0.82–1.87)	NA	1.22 (0.81–1.85)
Peutz-Jeghers syndrome	NA	2.14 (0.69–6.65)	NA	2.14 (0.69–6.65)
Annual income (US dollars)				
*<50,000*	NA	1.00 (ref)	NA	1.00 (ref)
*50,000–75,000*	NA	0.88 (0.69–1.12)	NA	0.89 (0.69–1.13)
*75,001–100,000*	NA	1.02 (0.79–1.33)	NA	1.05 (0.81–1.36)
*>100,000*	NA	1.06 (0.86–1.32)	NA	1.10 (0.89–1.36)
Neighbourhood-level factors				
Percentage of high school graduates				
*Quintile 5 (high)*	NA	NA	1.00 (ref)	1.00 (ref)
*Quintile 1 (low)*	NA	NA	0.79 (0.58–1.08)	0.81 (0.60–1.11)
*Quintile 2*	NA	NA	0.95 (0.67–1.36)	0.94 (0.66–1.33)
*Quintile 3*	NA	NA	1.16 (0.77–1.75)	1.16 (0.77–1.76)
*Quintile 4*	NA	NA	1.04 (0.67–1.60)	1.12 (0.72–1.73)
Percentage of assisted income				
*Quintile 1 (low)*	NA	NA	1.00 (ref)	1.00 (ref)
*Quintile 2*	NA	NA	1.21 (0.94–1.56)	1.25 (0.97–1.62)
*Quintile 3*	NA	NA	1.15 (0.87–1.53)	1.18 (0.89–1.57)
*Quintile 4*	NA	NA	1.11 (0.82–1.52)	1.21 (0.89–1.66)
*Quintile 5 (high)*	NA	NA	1.16 (0.79–1.68)	1.33 (0.91–1.95)
Percentage with no health insurance				
*Quintile 5 (high)*	NA	NA	1.00 (ref)	1.00 (ref)
*Quintile 1 (low)*	NA	NA	0.79 (0.61–1.02)	0.84 (0.65–1.09)
*Quintile 2*	NA	NA	1.02 (0.76–1.36)	1.04 (0.77–1.40)
*Quintile 3*	NA	NA	0.91 (0.60–1.37)	0.97 (0.64–1.47)
*Quintile 4*	NA	NA	1.01 (0.64–1.61)	1.07 (0.67–1.72)
Percentage of vacant houses				
*Quintile 1 (low)*	NA	NA	1.00 (ref)	1.00 (ref)
*Quintile 2*	NA	NA	0.81 (0.63–1.05)	0.80 (0.62–1.03)
*Quintile 3*	NA	NA	0.83 (0.63–1.10)	0.77 (0.58–1.03)
*Quintile 4*	NA	NA	1.18 (0.90–1.55)	1.14 (0.87–1.51)
*Quintile 5 (high)*	NA	NA	0.81 (0.58–1.13)	0.76 (0.54–1.05)
Region				
*Mid-West*	NA	NA	1 (ref)	1 (ref)
*Northeast*	NA	NA	1.19 (0.95–1.50)	1.21 (0.96–1.52)
*South*	NA	NA	0.78 (0.57–1.09)	0.80 (0.58–1.12)
*West*	NA	NA	1.00 (0.73–1.37)	0.97 (0.70–1.33)

aHR – adjusted hazard ratio, CI – confidence interval, IQR – interquartile range, Mdn – median, NA – not applicable, ref – reference

*Model 1 was the null model; model 2 included individual-level factors; model 3 included neighbourhood-level factors; model 4 included individual- and neighbourhood-level factors. We excluded a history of progesterone-only prescribing from the primary models because we cannot distinguish therapeutic from contraceptive use. We report it in a separate sensitivity analysis (Table S4 in the **Online Supplementary Document**).

### Measures of random effects

The null model showed modest geographic clustering at both levels (**Table 3**). The state-level variance reached 0.0484, with a VPC of 2.8%, whereas the neighbourhood-level variance reached 0.0433, with a VPC of 2.5%. We calculated both VPCs using the random effects and the extreme-value residual variance of π^2^/6 in the denominator. Individual-level covariates increased state-level variance by 16.7%, from 0.0484 to 0.0565, and reduced neighbourhood-level variance by only 10.4%, from 0.0433 to 0.0388. These changes indicated that population composition explained little of the observed geographic variation. Neighbourhood-level covariates reduced both variance components to approximately 10^−5^ to 10^−6^, which placed them at the boundary of the parameter space. Removing the region term did not restore either variance component.

### Sensitivity analysis

Of the 778 incident cases, we identified 165 (21.2%) when using a single recorded EC concept, 613 when we required at least two concepts, and 500 when we required at least three concepts. We found that these stricter case definitions strengthened the association with obesity, with aHRs increasing from 2.16 to 2.21 and 2.41, respectively, and with diabetes, where the aHRs increased from 1.27 to 1.34 and 1.37. We also observed an increase in aHR comparing non-Hispanic White and non-Hispanic Black women from 1.44 to 1.63 and 1.71. In contrast, we observed the Northeast–Midwest aHR moved towards the null, from 1.21 to 1.03 and 0.90. We found that state-level variance increased from effectively zero to 0.055 and 0.141, while VPC increased from 0.0% to 3.2% and 7.8%. All three variance components were imprecisely estimated (Table S1 in the **Online Supplementary Document**).

We identified obesity as the only covariate that violated the proportional hazards assumption. We detected this violation using the Schoenfeld test (*P* = 0.008) but found no violation using the global test (*P* = 0.28). We observed a stronger association in later follow-up (aHR = 2.45; 95% CI = 1.98–3.02 after the first three years) than during the first three years (aHR = 1.90; 95% CI = 1.55–2.35). We found that the interaction with the follow-up period did not reach conventional statistical significance (*P* = 0.087) (Table S4 in the **Online Supplementary Document**).

We excluded women with a recorded prior hysterectomy at cohort entry and reported EC incidence among women with an intact uterus. We recorded prior hysterectomy exclusion prevalence of 4.5% among non-Hispanic White women and 5.1% among non-Hispanic Black women (Table S6 in the **Online Supplementary Document**). Previous research reported corresponding prevalence estimates of 15.6% and 16.3% [21].

We added a history of progesterone-only prescribing to the model. The exposure group included 1,592 women and 27 exposed events. We found that this variable did not alter any other estimate. The obesity hazard ratio changed from 2.156 to 2.163, the diabetes estimate remained unchanged at 1.267, the race estimate changed by 0.002, and the region estimate changed by 0.007. We found no statistically significant association between progesterone-only prescribing and EC incidence (aHR = 1.46; 95% CI = 0.98–2.18). We calculated E-values of 5.39 for women aged 60–69 years, 3.74 for obesity, and 2.24 for the comparison of non-Hispanic White with non-Hispanic Black women (Table S7 in the **Online Supplementary Document**).

## DISCUSSION

We used the largest nationally representative longitudinal data from the USA to identify the factors predicting EC. The large cohort, area-level contextual data, and number of incident EC cases allowed us to apply mixed-effects methods to model development.

We observed an age-related increase in EC risk: compared with women younger than 40 years, women aged 40–49, 50–59, 60–69, and 70 years or older had 2.4, 2.9, 3.0, and 2.9 times the risk of EC, respectively. The significantly increased risk from age 40 aligned with increases in EC incidence during perimenopause and after menopause that previous studies reported in different populations [1,22,23]. This finding also aligns with the established role of unopposed oestrogen exposure resulting from relative progesterone deficiency in endometrial carcinogenesis [24]. Irregular ovulation during perimenopause may increase EC risk and lead to unopposed oestrogen accumulation, a major driver of endometrial thickening [25].

We observed a nonlinear association between age and EC risk, with risk peaking among women aged 60–69 years rather than increasing continuously. Hormonal shifts, lifestyle, and timing of menopause may explain this pattern, as this peak coincides with the cessation of ovulation and the highest prevalence of risk factors like obesity, diabetes, and metabolic syndrome [26,27], while biological changes and competing risks, including survival bias and deaths from other age-related diseases, may explain the subsequent decline [28]. The complex age patterns we observed require investigation through longer longitudinal studies that would use competing risk analyses to account for mortality from other causes and examine how the association between age and EC varies across populations and time periods. Life-course epidemiological approaches should examine how reproductive patterns, occupational exposures, and healthcare utilisation throughout women's lives contribute to EC risk across age groups.

We also observed a statistically significant association of race and ethnicity with increased EC risk. Non-Hispanic White women had a higher incidence of EC than non-Hispanic Black women in this cohort. We excluded women with a recorded prior hysterectomy at cohort entry and therefore estimated incidence among women with an intact uterus in each group. We captured hysterectomy in AoU only when it was captured in the EHR or survey responses. Previous studies have shown that prophylactic and therapeutic total abdominal hysterectomy, with or without bilateral salpingo-oophorectomy, reduces the risk of developing EC, especially EC associated with Lynch syndrome, which is more common in people of African ancestry than in other ethnic groups [29,30]. Under-ascertainment would most strongly affect non-Hispanic Black women because this group has a substantially higher true prevalence of hysterectomy. Incomplete exclusion would inflate their at-risk denominator and lower their apparent incidence, which corresponds to the direction of the difference we observed. We therefore reported exclusion counts by race and compared the observed exclusion prevalence with published estimates.

The observed difference in incidence should not be interpreted as evidence of a lower overall burden of disease among non-Hispanic Black women. These women consistently experience worse outcomes after an EC diagnosis, reflecting differences in tumour biology, stage at diagnosis, access to specialist care, and treatment receipt. Future studies should examine EC incidence and mortality simultaneously to avoid misinterpreting lower EC incidence among Black women as evidence of lower overall burden [31].

The lower incidence of EC observed among non-Hispanic Black women a critical research priority that could inform prevention strategies for other populations. Future studies should integrate genetic analyses, detailed reproductive histories, investigations of dietary patterns, and measures of healthcare utilisation to examine possible biological, behavioural, social, and healthcare-related explanations. Mechanistic research should also investigate whether protective factors identified in non-Hispanic Black populations can be translated into interventions for other ethnic groups.

Our finding that obesity was associated with a higher EC risk is supported by the results of similar studies [32,33]. The mechanisms linking obesity to endometrial carcinogenesis include peripheral aromatization of androgens to oestrogens in adipose tissue, hyperinsulinemia-driven endometrial proliferation, and chronic low-grade inflammation that promotes genomic instability [8,34,35]. Potential strategies for reducing EC risk in individuals with obesity include weight loss through dietary changes, exercise, and bariatric surgery. These strategies also include pharmaceutical interventions such as metformin, glucagon-like peptide-1 receptor agonists, and progestins to manage metabolic factors [34,35].

Consistent with prior epidemiological studies [36–38], we found that diabetes was associated with higher EC risk. Hyperinsulinemia, which commonly occurs in diabetes, could be the biological mechanism underlying this association. Insulin has been shown to stimulate the growth of endometrial stromal cells by binding to insulin receptors on endometrial cells, leading to proliferation [39–41].

Our analysis suggests that measured individual- and neighbourhood- level factors explain apparent state-level clustering. Policymakers should nevertheless consider state-level interventions that could address EC disparities, including standardising gynaecologic oncology referral networks across states to ensure equitable access to specialised care; implementing uniform cancer registry reporting standards to improve surveillance accuracy; coordinating Medicaid expansion and coverage policies that affect access to cancer screening; and developing state-specific cancer prevention programmes tailored to demographic and socioeconomic profiles identified in multilevel analyses.

Research on healthcare infrastructure should systematically examine the relationships among specialist availability, imaging centre density, and apparent cancer incidence rates to distinguish access effects from true aetiological differences. Such research could inform healthcare policy and resource allocation, particularly efforts to address potential disparities in access to cancer care across regions and communities.

Future studies should systematically examine specific state-level factors that may influence EC incidence and detection. These factors should include Medicaid expansion status and timing, which affect healthcare access for low-income women and may influence cancer detection rates. Researchers should also examine the density and geographic distribution of gynaecologic oncologists, which could affect detection and referral patterns, and state cancer registry completeness and reporting requirements, which may influence apparent incidence rates. Future studies should also assess state-specific insurance regulations and coverage mandates for cancer screening, public health infrastructure, and funding levels for cancer prevention programmes. Moreover, they should incorporate measures of healthcare infrastructure, including gynaecologic oncology specialist density, imaging centre availability *per capita*, cancer screening rates, and regional diagnostic testing patterns, to distinguish surveillance bias from true aetiological differences in regional cancer patterns.

Finally, more research is needed into how epidemiological findings can be effectively translated into clinical practice guidelines and public health policies. This includes studying barriers to implementing risk-stratified screening approaches, evaluating the effectiveness of targeted interventions for high-risk demographic groups, and examining how healthcare system changes can improve equity in cancer detection and care across populations and geographic regions.

### Study limitations

Our study has several limitations that should be considered when interpreting the findings. First, we used ZIP codes as neighbourhood proxies because of data availability in the AoU database, which represents a significant limitation. Smaller geographic units, such as census tracts or block groups, would provide more precise neighbourhood definitions, but we could not link these units to our cohort data. Future epidemiological research should prioritise the development of more sophisticated geographic analysis methods that can better capture true neighbourhood effects on cancer risk.

Second, we could not differentiate between type 1 and type 2 diabetes when examining EC risk. Type 2 diabetes is more prevalent than type 1 diabetes, so most diabetes cases in this study likely involved type 2 diabetes. Future studies should consider stratifying their analysis by diabetes type.

Third, the AoU cohort requires participants to have had at least one clinical visit before and after the index date. This requirement introduced healthy volunteer bias and systematically excludes women with limited healthcare engagement, a group likely to include women at the highest socioeconomic risk. This bias may underestimate true neighbourhood-level effects on EC incidence and attenuate associations with insurance and income variables.

Fourth, the healthcare engagement requirement may have also introduced a detection bias because greater clinical contact increases the likelihood of an EC diagnosis. This bias may inflate incidence estimates among groups with higher socioeconomic status.

Fifth, AoU does not record established protective hormonal exposures, particularly combined oral contraceptive use, so we could not include them in our analysis.

Lastly, the AoU does not provide data on key reproductive history variables, including parity, age at menarche, and age at menopause. These variables are established EC risk factors, and their omission may have introduced residual confounding in the multivariable models.

## CONCLUSIONS

We found that individual-level characteristics mainly drove EC incidence in the USA and identified age and obesity as the strongest independent predictors. The approximately 3-fold increased risk among individuals aged 60–69 years and 2-fold increased risk among individuals with obesity make these groups prime candidates for targeted surveillance and intervention.

## Additional material


Online Supplementary Document

## Data Availability

**Data availability:** We obtained the data used in this study from the National Institutes of Health’s All of Us Research database and the American Community Survey, which are publicly available for use. The All of Us Research Program has established procedures for accessing its data. We will direct interested parties to the appropriate contacts to request access and provide our analytical methods upon request.
