# Supplementary material for: Risk factors for endometrial cancer incidence and regional and ethnic disparities in the USA
Source: J Glob Health. 2026 Oct 23;16:04368. doi: 10.7189/jogh.16.04368 (PMC13693290; doi:10.7189/jogh.16.04368)
Supplement: Supplementary file 1 [file jogh-16-04368-s001.pdf]

Supplement to: Adekanmbi V, Guo F, Wang J, Zhang A, Wang CH, Hoang TN, Sokale I, Uthman O, Berenson AB. Risk factors for endometrial cancer incidence and regional and ethnic disparities in the USA. J Glob Health. 2026;16:04368.

Supplemental materials

Table R1. Principal estimates before and after the cohort refresh.

Estimate	Submitted version	Revised (refreshed cohort)
Eligible women	84,887	81,171
Incident EC cases	823	778
Person-years	~508,600	496,709
Obesity, aHR	2.11 (1.82–2.45)	2.16 (1.85–2.52)
Diabetes, aHR	1.24 (1.04–1.47)	1.27 (1.06–1.51)
NH White vs NH Black, aHR	1.42 (1.14–1.77)	1.44 (1.15–1.80)
Northeast vs Midwest, aHR	1.29 (1.03–1.62)	1.21 (0.96–1.52)

Table R2. Effect estimates and variance components under progressively stricter case definitions.

Estimate	≥1 code (778)	≥2 codes (613)	≥3 codes (500)
Obesity	2.16 (1.85–2.52)	2.21 (1.86–2.63)	2.42 (2.00–2.95)
Diabetes	1.27 (1.06–1.51)	1.34 (1.10–1.64)	1.37 (1.10–1.70)
NH White vs NH Black	1.44	1.63	1.71
Northeast vs Midwest	1.21 (0.96–1.52)	1.03 (0.68–1.55)	0.90 (0.51–1.58)
State-level variance	0.00002	0.0547	0.1409
VPC, state (%)	0.00	3.22	7.83

Table R3. Variance components across the model ladder, with and without the region term.

Model	State var.	Δ state	ZIP var.	Δ ZIP	AIC
M1 null	0.0484	ref	0.0433	ref	17,411.9
M2 individual only	0.0565	+16.7%	0.0388	−10.4%	17,212.0
M3 area-level only	0.00002	−100%	0.00002	−100%	17,419.0
M4 full, no region	0.000002	−100%	0.000003	−100%	17,221.2
M4 full (published)	0.00002	−100%	0.00002	−100%	17,222.4

Table R4. Obesity hazard ratio by period of follow-up (interaction LRT p = 0.087).

Follow-up period	Events	Obesity aHR (95% CI)
0–3 years	387	1.90 (1.55–2.35)
>3 years	391	2.45 (1.98–3.02)

Table R5. Effect estimates with and without the progesterone-history covariate.

Estimate	Without progesterone	With progesterone
Obesity	2.156 (1.848–2.515)	2.163 (1.854–2.524)
Diabetes	1.267 (1.061–1.514)	1.267 (1.061–1.514)
NH Black vs NH White	0.694 (0.554–0.868)	0.696 (0.556–0.871)
Northeast vs Midwest	1.209 (0.958–1.524)	1.216 (0.964–1.534)
History of progesterone use	—	1.464 (0.984–2.176)

Table R6. Hysterectomy exclusion counts stratified by race/ethnicity.

Race/Ethnicity	N	N_hyst	pct
Non-Hispanic Black	18,172	925	0.0509
Hispanic	1,541	56	0.0363
Other/Unknown	2,868	101	0.0352
Non-Hispanic White	58,590	2,662	0.0454

N is the sample size in our final cohort (after excluding hysterectomy), n_hyst is the number of patients excluded

Table R7. E-values for the principal effect estimates.

Estimate	aHR (95% CI)	E-value, point	E-value, CI limit
Age 60–69 vs <40	2.97 (2.21–4.00)	5.39	3.85
Obesity	2.16 (1.85–2.52)	3.74	3.10
NH White vs NH Black	1.44 (1.15–1.80)	2.24	1.57
Diabetes	1.27 (1.06–1.51)	1.86	1.31
Northeast vs Midwest	1.21 (0.96–1.52)	1.71	1.00
